# The Throat and Nose and Their Diseases

**Published:** 1900-06

**Authors:** 


**The Throat and Nose and their Diseases.** By LENNOX BROWNE. Fifth Edition. Pp. 967. London: Baillière, Tindall & Cox. 1899.

This, the fifth edition of a work which first appeared twenty-one years ago, more than maintains the increased dimensions which the author has found necessary with each edition as compared with its predecessors, an increase due not only to the almost daily expansion of laryngology, but also to the inclusion in its territory of the equally widening fields of rhinology. Mr. Browne has added detailed information on the normal anatomy, physiology, and histology, as well as on morbid anatomy and bacteriology, while the chapters on tonsillitis, diphtheria, tuberculosis, lupus, leprosy, malignant neoplasms, and on nervous diseases, have been largely re-written. Separate chapters, too, are accorded to diseases of the lingual tonsil, of the pharyngeal tonsil, and of the accessory nasal cavities.

The author has received valuable assistance from Mr. Mayo Collier on the subject of anatomy, and from the late Dr. Cagney on nervous diseases, while for the details of normal and morbid anatomy Mr. Wyatt Wingrave has been responsible, the text being freely illustrated by a series of excellent reproductions from his microscopical sections.

On several questions the author's views widely differ from those of the majority of experienced laryngologists. Thus, for instance, he dwells at some length on the subject of varix of the tongue and hypertrophy of the lingual tonsil, and states that "in no less than 438 cases, note was taken of a varying degree of venous congestion and fulness, which he generically terms lingual varix, in a total of 1547 patients suffering from diseases of the throat and nose" in his private practice. This varicose state was, of course, of very variable grade, and the term is used by the author to signify any excess of venous engorgement. We fear that the undue prominence given to these conditions which are so constantly present in the majority of individuals past middle life, without giving rise to any throat symptoms, is apt to induce inexperienced readers to employ the galvano-cautery with undesirable frequency and cause unnecessary suffering, notwithstanding the author's terse dismissal of the warning of other recent writers against the risks in this method of treatment.

Mr. Browne is not a strong believer in the value of the anti-toxin treatment of diphtheria, and while pointing out possible disadvantages that attend its use, he says very little in support of the serum which in the experience of the vast majority of practitioners has yielded such excellent results.

The author modifies his former views on the question whether "benign growths occasionally assume a malignant and even a cancerous character by the irritation produced by attempts at removal," and with commendable fairness cites the collected statistics which have rendered his own opinion so difficult to maintain. Mr. Browne recognises the work of other writers and practitioners in the subjects included in his treatise, while clearly and fully setting forth his own views. The section on diseases of the nasal accessory sinuses, clear and accurate as far as it goes, is rather meagre, and lacking in the detail one might fairly expect in this large volume.

Though this book is somewhat overweighted with general pathology having no special bearing on throat diseases, it will be valued especially as a work of reference, while the fulness and, in some sections, discursive character of the text will be appreciated by the advanced student.

The illustrations throughout the work, both coloured and in monochrome, are very numerous and beautiful, and the author's artistic skill cannot fail to give pleasure as well as valuable instruction to the reader; indeed, it may be truly said that the illustrations alone are worth the cost of the volume before us.

